# Believing What You're Told: Politeness and Scalar Inferences

**DOI:** 10.3389/fpsyg.2018.00908

**Published:** 2018-06-13

**Authors:** Diana Mazzarella, Emmanuel Trouche, Hugo Mercier, Ira Noveck

**Affiliations:** ^1^Leibniz-Zentrum Allgemeine Sprachwissenschaft (ZAS), Berlin, Germany; ^2^Cognition and Development Lab, Department of Psychology, Yale University, New Haven, CT, United States; ^3^Institut Jean Nicod, Centre National de la Recherche Scientifique, Paris, France; ^4^Institut des Sciences Cognitives Marc Jeannerod, Centre National de la Recherche Scientifique, Bron, France

**Keywords:** experimental pragmatics, scalar inference, some, face, politeness, epistemic vigilance

## Abstract

The experimental pragmatics literature has extensively investigated the ways in which distinct contextual factors affect the computation of scalar inferences, whose most studied example is the one that allows “Some *X*-ed” to mean *Not all X-ed*. Recent studies from Bonnefon et al. (2009, 2011) investigate the effect of politeness on the interpretation of scalar utterances. They argue that when the scalar utterance is face-threatening (“Some people hated your speech”) (i) the scalar inference is less likely to be derived, and (ii) the semantic interpretation of “some” (*at least some*) is arrived at slowly and effortfully. This paper re-evaluates the role of politeness in the computation of scalar inferences by drawing on the distinction between “comprehension” and “epistemic assessment” of communicated information. In two experiments, we test the hypothesis that, in these face-threatening contexts, scalar inferences are largely *derived* but are less likely to be *accepted as true*. In line with our predictions, we find that slowdowns in the face-threatening condition are attributable to longer reaction times at the (latter) epistemic assessment stage, but not at the comprehension stage.

## Introduction

Scalar inferences are classically described as pragmatic enrichments (made by a listener) when a speaker uses a weaker term (e.g., “some”) to communicate a narrowed, more informative, meaning that excludes a stronger term (e.g., “all”). Consider the following example:

(1)
  - Some students failed the exam.
  - Not all of the students failed the exam.

While the semantic (encoded) meaning of “some” is compatible with *all*, (1a) is frequently interpreted as communicating (1b). The inference from (1a) to (1b) is called *scalar* because, based on earlier accounts (e.g., Horn, 1984), the enrichment exploits an implicit scale of informativeness that ranges from *some* to *all*. The explanation goes as follows: the addressee assumes that the speaker would have said “all” if she thought the statement with “all” was true; the choice of “some” thus implies either that she does not know whether *all* is the case or that she believes *all* is not the case. If it is reasonable to assume that the speaker knows whether the stronger alternative holds or not, the use of a relatively weak expression is taken to indicate that the speaker believes the stronger alternative to be false.

Scalar inferences have become the drosophila of experimental pragmatics as they have the means to provide a clear test case to investigate the interaction of semantics with contextual information in sentence processing. The way the scalar enrichment is carried out has been vigorously debated in this experimental literature (see Chemla and Singh (2014a,b) and Noveck (2018) for recent reviews). On one side are those who argue that scalar inferences occur routinely and independently of context, i.e., by default (Levinson, 2000). On the other side are those who defend context-sensitivity and argue that such enrichments occur as a function of particular features of a task or conversational context and that these particular instances need not arise with the mere expression of a weak scalar term (see Sperber and Wilson, 1986/1995; Carston, 1998, but also Chierchia (2013) for a discussion of context-sensitivity within a grammatical framework). Much evidence supports the latter characterisation. As has been reported and summarized elsewhere (Noveck and Sperber, 2007; Noveck and Reboul, 2008; Noveck and Spotorno, 2014), linguistically encoded readings are often sufficient for making on-line interpretations with utterances containing weak scalar terms.

Recent research has looked into the time course of the derivation of scalar inferences in real-time language comprehension. Many studies report that enriched readings (e.g., *some but not all*) are linked with the availability or application of supplementary processing (e.g., see Bott and Noveck, 2004; Breheny et al., 2006; De Neys and Schaeken, 2007; Huang and Snedeker, 2009; Bott et al., 2012). However, there is disagreement in the literature concerning the source of these slowdowns (see Foppolo and Marelli, 2017). Moreover, it appears that the speed at which a scalar inference is computed may depend on features of the context of utterance, such as the naturalness and availability of alternatives (Breheny et al., 2013; Degen and Tanenhaus, 2015).

The debate concerning the kinds of contextual factors that affects the computation of scalar inferences has been recently enriched by the work of Bonnefon and colleagues. This work investigates the effect of politeness on the derivation of scalar inferences and it presents a case for the following two claims. First, politeness is likely to block the computation of the scalar inference. Second, the unenriched interpretation of the scalar utterance in politeness contexts requires supplementary processing costs (Bonnefon et al., 2009, 2011; Feeney and Bonnefon, 2012). The aim of this paper is to address these two claims by (i) assessing the robustness of Bonnefon and colleagues' results, and by (ii) providing a finer-grained analysis of the processing of scalar utterances in these experiments. This analysis will be based on the distinction between “comprehension” and “epistemic assessment” (see, Mazzarella, 2015), and will ultimately describe Bonnefon and colleagues” results as linked to the process of “epistemic assessment” (with no direct bearing on the comprehension of the scalar utterance).

## Face-threatening contexts and scalar inference

In a series of studies, Bonnefon and colleagues investigated the derivation of scalar inferences in face-threatening contexts. These are situations in which the public image or positive identity of the addressee is threatened (Brown and Levinson, 1987). For instance, consider the following example:

(2)
  - Some people hated your speech.
  - Not all the people hated your speech.

The scalar utterance (2a) carries a threat toward the public image of the addressee (represented by lack of public approval and support). Because of this, Bonnefon and colleagues argue, the addressee would be less likely to derive the scalar inference (2b) and would take the use of “some” as a polite device adopted by the speaker to sugar-coat the information conveyed. Specifically, they claim that “face-threatening contexts make the narrowed interpretation of “some” less appropriate” and report that, in line with this, their “result suggests that people's tendency to draw the scalar inference from “some *X*-ed” to “not all *X*-ed” decreases when *X* threatens the face of the listener” (Bonnefon et al., 2009, p. 250–251).

The empirical support for these claims comes from a series of similar studies where the authors investigate the interpretation of face-threatening and face-boosting scalar utterances. While the former carry a threat toward the “face” of the addressee, the latter reinforce his positive identity. They present participants with short vignettes in which they are asked to imagine to have carried out a publicly observed act (such as giving a speech in front of a small group of people). Critically, participants are provided with feedback in the form of a scalar utterance. The feedback is negative in the face-threat condition (“Some people hated your speech”) and positive in the face-boost one (“Some people loved your speech”). This is followed up with a meta-linguistic question about the feedback. The entire task can be broken down into three parts: the task's background information (3), the scalar utterance (4), and what we call the *semantic compatibility question* (5), which prompts the critical “Yes” or “No” responses:

(3) Imagine you gave a speech at a small political rally. You are discussing your speech with Denise, who was in the audience. There were 6 other people in the audience. You are considering whether to give this same speech to another audience.
(4) Hearing this, Denise tells you that “Some people hated your speech.”
(5) Given what Denise told you, do you think that it is possible that everybody hated your speech?

Importantly, the semantic compatibility question is the measure used to determine whether or not a scalar inference has been derived. A “No” answer is taken to suggest that participants have derived the scalar inference (hence the perceived incompatibility between what Denise said and the state of affairs in which *everybody* hated/loved the speech). On the contrary, a “Yes” answer is taken to reveal that participants have adopted the semantic interpretation of “some,” *at least some and possibly all*, which is consistent with the possibility that *everybody* hated/loved the speech.

Bonnefon et al. (2009, 2011) have consistently found that the percentage of participants answering “Yes” is significantly higher in the face-threat condition than it is in the face-boost condition (see Table 1). That is, participants are more likely to think that it is possible that everybody *hated* the speech—after being told that “some” did—than to think that everybody *loved* the speech when similarly told that “some” did.

**Table 1:** Percentage of “Yes” responses to the semantic compatibility question.

	**Face-threat (%)**	**Face-boost (%)**
Bonnefon et al. (2009)	42	17
Bonnefon et al. (2011)	55	27

*For each cell, the complement corresponds to “No” responses*.

Bonnefon et al. (2011) went further by measuring response times, which covered the scalar utterance (4) *and* the time to answer the semantic compatibility question (5). They reported that response times were longer when participants answered “Yes” as opposed to “No,” but only in the face-threat condition (the interaction was significant at the 0.5 level using one-tailed tests). The authors concluded that in face-threatening contexts the semantic interpretation of “some” is associated with extra-processing effort. In light of this, Bonnefon et al. (2011, p. 3393) argue that politeness “appears to add a layer of complexity to the usual processes involved in the interpretation of “some.”” This layer of complexity would make the semantic or “broad” interpretation of “some” the more effortful one.

To summarize, Bonnefon and colleagues put forward the following two claims: (i) scalar inferences are less likely to be derived in face-threatening contexts; (ii) the semantic interpretation of face-threatening scalar utterances involves an extra cognitive cost. Taken together, these two claims are presented as an interesting challenge to current cognitive models of scalar inference. Specifically, they target the assumption that semantic interpretations are always less effortful to arrive at than pragmatic interpretations: “Showing that face-threatening contexts encourage broad interpretations whilst making them harder would require to revisit this basic assumption.” (Bonnefon et al., 2011, p. 3390).

In what follows, we take the following three steps. First, we analyse the structure of Bonnefon et al.'s task and describe a confound that undermines their main claim. Once this confound is exposed, it becomes arguable that the two interpretations linked to the existential quantifier “some” are not themselves the source of the exceptional results. Second, we consider a relatively new line of research that distinguishes between “comprehension” and “epistemic assessment” of the communicated content (Sperber et al., 2010) and discuss its implications for the role of politeness on the processing of scalar utterances. Finally, we introduce our experiments which aim at disentangling the interpretation of the scalar utterance from the participant's evaluative task.

## Comprehension and epistemic assessment: a methodological confound and a theoretical conflation

The starting point of our critical discussion involves a closer analysis of Bonnefon and colleagues' original paradigm, and especially the (2011) follow-up paper that further presented reaction times. We focus on (i) the way reaction times were collected, and, (ii) the nature of the test question (i.e., the semantic compatibility question).

To start, it is crucial to notice that, in Bonnefon et al.'s (2011) study, the scalar utterance and the semantic compatibility question, (4) and (5) above, were displayed together on the screen as a block of text. The critical reaction time thus measured a (long) interval in which participants read the block *and* answered the question: the response time measure began with the advancing of the visual display of the text to “Hearing this, Denise tells […]” and ended when the answer key was pressed. Despite the length of the block and the fact that, arguably, two tasks are involved—reading the utterance and providing a Yes/No answer to the semantic compatibility question—the interaction effect is described as depending on the interpretation of the scalar utterance alone. Bonnefon et al. conclude that the semantic interpretation of “some” is derived more slowly than the pragmatic one, and thus that politeness increases the processing effort required to arrive at the semantic interpretation.

Furthermore, Bonnefon et al. see a direct link between responses to the semantic compatibility question (e.g., *[…] do you think that it is possible that everybody hated your speech?*) and the interpretation of the scalar utterance, with “Yes” answers corresponding to a semantic interpretation of “some,” and “No” answers to a pragmatic one. However, as discussed by Mazzarella (2015), the semantic compatibility question relates to the participant's *belief* that a certain state of affairs is likely to hold. It is *not* inherently a question about the speaker's informative intention (about the *interpretation* of the scalar utterance). In Bonnefon et al.'s task, the participant is asked to evaluate the likelihood concerning a state of affairs, e.g., that everybody hated the speech, when told earlier that “some” did. Note that the answer to the semantic compatibility question is not entirely dependent on the *interpretation* of the scalar utterance: participants could interpret it as communicating the scalar inference *Not all the people hated your speech*, and yet end up believing that it is possible that everyone indeed hated the speech. This is because the comprehension of a speaker's intended meaning and one's acceptance of it do not always go hand in hand. That is, an audience can *understand* a speaker's communicated content, e.g., that *not everyone hated the speech*, without *believing* it. Whether or not a listener accepts the incoming information depends on the plausibility of this information as well as on the trust the listener grants the communicator (Sperber et al., 2010).

The above discussion points toward the distinction between “comprehension” and “epistemic assessment”: while the former process relates to the interpretation of a piece of communicated information, the latter determines whether we believe it. This distinction has a long tradition in philosophy of language at least since the work of Austin (1962/98). Austin (1962/98) distinguishes between “securing the uptake” of an utterance, that is, comprehending its meaning and illocutionary force, from the utterance's perlocutionary effects. The latter comprise a range of cognitive and behavioral effects, “effects upon the feelings, thoughts, or actions of the audience” (Austin, 1962/98, p. 11), which go beyond uptake. These include the beliefs the audience forms with respect to what is communicated. Crucially, the latter may differ from the beliefs the communicator intended to induce in the audience.

This distinction between comprehension and epistemic assessment is overlooked in Bonnefon and colleague's work, as it is in the experimental pragmatics literature more generally. Instead of evaluating a participant's answer as a measure of accepting/rejecting the speaker's implied meaning, Bonnefon and colleagues directly map “Yes” answers from the semantic compatibility question to a semantic *interpretation* of “some” and “No” answers from the same question to a pragmatic *interpretation* of “some.” Furthermore, they explain the reaction time data as linked to the processes involved in the interpretation of the scalar utterance (“comprehension”), with no role assigned to epistemic assessment.

In light of the cognitive distinction between comprehension and epistemic assessment, Mazzarella (2015) proposes an alternative explanation of the data. She suggests that face-threatening contexts reduce the perceived honesty of the speaker and, as a result, decrease the likelihood of accepting the scalar inference as true. Mazzarella's (2015) hypothesis accounts for the higher percentage of “Yes” answers in the face-threat condition by suggesting that these answers reflect a rejection of the scalar inference. This possibility is not only theoretically plausible, but also empirically grounded. From a theoretical perspective, it is plausible to assume that the attribution of politeness concerns to the speaker might negatively affect the likelihood of her sharing some face-threatening information. If the speaker cared about saving the face of the addressee, she would try to minimize his face loss. In doing this, she could decide to withhold some relevant information, or even lie to the addressee^1^. If the addressee believes that this is the case, he might consider a polite speaker as less reliable from an epistemic point of view. Crucially, this hypothesis receives some support from two studies ran by Bonnefon et al. (2009) themselves. In their Experiment 2, they showed that, when presented with situations in which the speaker is described as knowing that *all* (e.g., that all the people hated/loved the speech), participants judge it as more likely that the speaker would use the word “some” in face-threatening contexts (“Some people hated your speech”) than in face-boosting ones (“Some people loved your speech”). Furthermore, in a second rating study, they asked participants to rate how “accurate,” “considerate,” “honest,” and “nice” it was of the speaker to use the word “some” in a context in which the speaker knew that *some but not all* and in a context in which she knew that *all*. Crucially, in the *all* condition, the use of “some” was rated as inaccurate and dishonest in both face-threatening and face-boosting contexts, but nice and considerate only in face-threatening contexts. If Mazzarella's (2015) account is on the right track, longer reaction times associated with “Yes” answers would be better explained as linked to the process of epistemically *evaluating* the scalar inference, which is triggered by the presence of the test question in (5).

## The current experiments

The aim of the current experiments is two-fold. First, we aim to confirm the robustness of Bonnefon and colleagues' categorical results, irrespective of the confound described in the previous section. Second, we will collect reaction time measures while addressing this confound in order to better determine how participants interpret the scalar utterance and the semantic compatibility question. That is, we adopt the same experimental paradigm but separate the presentation of the scalar utterance, (4), from that of the semantic compatibility question, (5). This way we can distinguish between what we call “the comprehension stage” and the “the epistemic assessment stage” and measure reaction times from each part (RT_utterance_ and RT_question_) and combine them. By separating the scalar utterance from the test question, we can better isolate the source of reaction time effects. Study 1 relies on Bonnefon et al.'s (2011) original materials, while Study 2 introduces some motivated changes to the materials in order to increase the likelihood that the scalar inference would be effectively derived.

### Study 1

In Study 1, we adopted Bonnefon et al.'s task but made two modifications. First, to better study the processes of deriving the scalar inference and of epistemically evaluating its factual plausibility, we separated the presentation of the scalar utterance and the semantic compatibility question. Second, we introduced a new question, which we refer to as the *conversational implicature question*: “Given what Denise tells you, do you think that she means that you should give the speech again to another group?” This question was presented after the semantic compatibility question, in order to preserve the integrity of Bonnefon et al.'s original task. See Appendix A for a thorough comparison between our materials and Bonnefon et al.'s (2011).

As a reminder, the first aim of Study 1 is to confirm Bonnefon et al.'s (2011) results. This would mean more “Yes” responses to the semantic compatibility question in the face-threat condition, as well as longer reaction times overall (for RT_utterance_ + RT_question_) for those responses when compared to the face-boost condition. Once this is accomplished, we will more carefully inspect reaction times to each part (RT_utterance_ and RT_question_).

As Bonnefon et al. (2011) maintain that slowdowns in the face-threat condition are linked directly to the way in which “some” is interpreted, they predict that the source of the expected interaction would be an observable difference between the face-threat and the face-boost conditions at the presentation of the scalar utterance. Specifically, they predict longer reaction time at the scalar utterance phase (i.e., RT_utterance_) for “Yes” answers than for “No” answers in the face-threat condition (with the latter being comparable with “Yes” and “No” answers in the face-boost condition). Following Mazzarella (2015), we predict that slowdowns will be observed for “Yes” answers uniquely at the presentation of the semantic compatibility question (for RT_question_), and not at the presentation of the scalar utterance (for RT_utterance_). Specifically, we predict longer reaction time at the question phase for “Yes” answers than “No” answers in the face-threat condition.

The aim of introducing the *conversational implicature question* is to determine the extent to which answers to the semantic compatibility question depend on the participants' understanding of the speaker's intention in the vignette. To understand why this is relevant, note that the speaker's utterance should be considered an indirect answer to the vignette's tacit question—should the addressee give the speech again? In the face-threatening version of this story, it is plausible that the addressee would take the utterance (*Some…hated*) as an indirect negative answer, which licenses the genuinely conversational implicature that the addressee should not give the speech again. Interestingly, this kind of implicature is warranted in the face-threat condition regardless of whether the scalar utterance is given a semantic interpretation—*At least some (and possibly all)* of the people hated your speech—or a pragmatic one—*Some but not all* of the people hated your speech. In both cases, the word “hated” (the face-threat condition) largely suffices for answering the indirect question regardless of one's concern for processing the word “Some”. Turning to the face-boosting context, the semantic and the pragmatic readings of the scalar utterance (*Some people loved your speech*) do interact differentially with the task's indirect question. If the utterance in the face-boosting context is interpreted as *You should not give the speech again* (which is plausible given Denise's tepid utterance) this judgment is consistent with an enriched reading of the scalar utterance (i.e., *only some people loved your speech so it is not clear that you should give the speech again*). On the other hand, if the scalar utterance is taken to implicate that *You should give the speech again*, it is more likely that the listener interpreted the existential quantifier as *Some and perhaps all*. Crucially, the interpretative path of the face-boosting utterance is bound up with the way it is interpreted.

It is worth noting that the negative valence of the verb “hate” may be stronger than the positive valence of the verb “love.” As a result, answers to the conversational implicature question may be more subject to individual variability in the face-boost condition than in the face-threat condition. As a result of this asymmetry between the face-boosting and the face-threatening stories, it is not clear whether they are equally likely to lead to the pragmatic enrichment of “Some”. By explicitly introducing the *conversational implicature question* in our task, we thus investigate this potential asymmetry between the face-threatening and the face-boosting context.

#### Material and methods

We recruited 399 participants through Amazon Mechanical Turk^2^ (228 men, 171 women, mean age 32.3, *SD* = 10.1). Each participant read the Speech vignette either in the face-boosting version or in the face-threatening one. The face-threating version of the Speech vignette read as follows:

(6a) Imagine you gave a speech at a small political meeting. You are discussing your speech with Denise, who was also there. There were 6 other people in the audience that day. You tell Denise that you are thinking about giving the same speech to another group.
(6b) Hearing this, Denise tells you that “Some people hated your speech.”

We made some minor adjustments to Bonnefon et al.'s task in order to make it clearer (e.g., it is more appropriate to call a gathering of six people a “meeting” rather than a “rally”). Texts corresponding to (6a) and (6b) were displayed in two separate screens. In the face-boost version, *Some people hated your speech* was replaced with *Some people loved your speech*. After reading the story, participants were asked the following questions (always in this order):

(6c) Given what Denise told you, do you think that it is possible that everybody hated [loved] your speech?
(6d) Given what Denise tells you, do you think that she means that you should give the speech again to another group?

These questions were followed by two options, “Yes” and “No.” Participants were required to click on one of them.

This study was carried out in accordance with the Décret n° 2017-884, whose article R. 1121- 1. -IId indicates that such research does not have to receive IRB approval in France. All subjects gave written informed consent in accordance with the Declaration of Helsinki.

#### Analysis

In order to retain the cleanest data possible, we first removed from our analysis participants who (i) unnecessary clicked on the relevant screen more than once^3^ (91 participants), as well as participants who (ii) showed a clear lack of attention during the task by exceeding the following reaction times: RT_UTTERANCE_ > 20 s, RT_QUESTION_ > 30 s (3 participants). Finally, we log transformed the data and we removed 13 participants as outliers using the criteria of 2.5 SD away from the mean for RT_utterance_ and for RT_question_. Our final sample included 292 participants.

#### Results

##### The semantic compatibility question

In the face-threat condition, 45% of participants answered “Yes” to the question “…do you think it is possible that everybody hated your speech?” This percentage dropped to 32% in the face-boost condition (Fisher exact test, *p* = 0.02, *OR* = 1.7). The difference across conditions, though slightly narrower than those reported by Bonnefon et al. (2009, 2011), replicates their original findings and confirms that participants are significantly more likely to respond positively to the semantic compatibility question in the face-threat condition than they are in the face-boost one (Table 2).

**Table 2:** Percentage of “Yes” responses to the semantic compatibility question and the conversational implicature question in Study 1.

	**Hearing this, Denise tells you that**	
	“Some people hated your speech” **Face-threat condition**	“Some people loved your speech” **Face-boost condition**
**SEMANTIC COMPATIBILITY QUESTION**		
do you think that it is possible that everybody hated [loved] your speech?	45%	32%
**CONVERSATIONAL IMPLICATURE QUESTION**		
do you think that she means that you should give the speech again to another group?	7%	64%

*For each cell, the complement corresponds to “No” responses*.

##### The conversational implicature question

When asked whether the speaker meant that the participant should give the speech again, the participants in the face threat condition were practically unanimous in saying “No”—only 7% responded with “Yes.” In the face-boost condition, participants were more divided: 36% said “No” and 64% answered “Yes.” This result confirms our prediction that the conversational implicature would vary across the two conditions.

In order to see how the semantic compatibility question is influenced by the conversational implicature (Table 2), we split participants according to their answers. Preserving the order of the questions, there are four possibilities: Yes-Yes, Yes-No, No-Yes, No-No. Tables 3, 4 provide the distribution of the participants in the face-threat and face-boost conditions respectively.

**Table 3:**
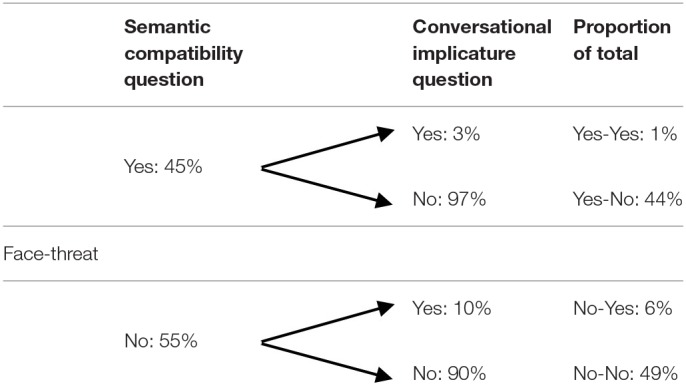
Percentage of “Yes”/”No” answers to the semantic compatibility question and the conversational implicature question in the face-threat condition (Study 1).

**Table 4:**
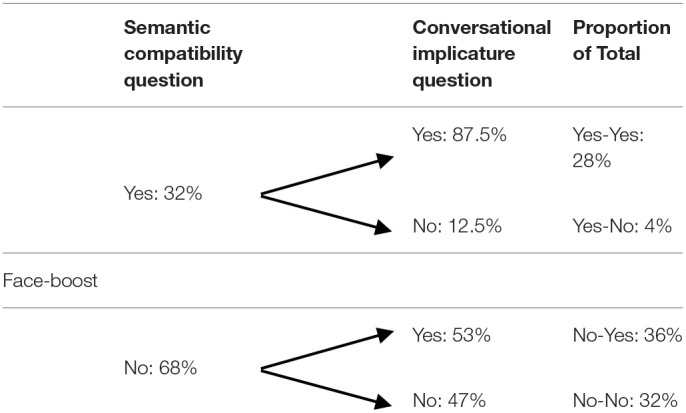
Percentage of “Yes”/”No” answers to the semantic compatibility question and the conversational implicature question in the face-boost condition (Study 1).

These data are particularly relevant with regard to the face-boost condition as they provide insight into how to interpret participants' answers to the semantic-compatibility question. As discussed above, a “No” answer to the conversational implicature question provides us with an indication about the scalar inference. That is, it is plausible to assume that those who carry out and adopt the scalar inference in the face-boost condition (to infer and commit to *Some but not all the people loved your speech* by saying “No” to the semantic compatibility question) are more likely to conclude *You should not give the speech again*. Arguably, one might not want to give a speech again if only a subset of the group loved the speech. This allows us to speculate about the pattern of answers for the group of participants who answered “Yes” to the conversational implicature question (*You should give the speech again*). Most likely, those who answer “Yes” to both questions in the face-boost condition represent a group of participants who have arguably not derived the scalar inference at all (28% of the total).

##### Reaction times

In the top of Table 5, we present the results by combining RT_utterance_ and RT_question_ in order to make them comparable to Bonnefon et al.'s (2011). A two-way ANOVA was conducted that examined the effect of Response Type and Face Condition (Face-boost/Face-threat) on the combined response times. The results do not strongly replicate Bonnefon et al. (2011). While our results are in line with their data, we only find a tendency toward an interaction, [*F*_(1, 288)_ = 2.75, *p* = 0.10], reflecting the fact that participants took longer to answer “Yes” but only in the face-threat condition. We did not find a main effect for Response Type [*F*_(1, 288)_ = 0.32, *p* = 0.57] nor for Face Condition [*F*_(1, 288)_ = 0.53, *p* = 0.46].

**Table 5:** Mean response time (in seconds) for “Yes” and “No” answers to the semantic compatibility question, in the face-boost and in the face-threat conditions (Study 1).

	**Face-threat**	**Face-boost**
**SCALAR UTTERANCE + SEMANTIC COMPATIBILITY QUESTION**		
“Yes”	8.2 s (2.6)	7.5 s (2.9)
“No”	7.6 s (2.7)	7.9 s (3.1)
**SCALAR UTTERANCE**		
“Yes”	3.0 s (1.0)	3.1 s (1.5)
“No”	2.9 s (1.3)	3.3 s (1.6)
**SEMANTIC COMPATIBILITY QUESTION**		
“Yes”	5.2 s (1.9)	4.4 s (2.1)
“No”	4.6 s (1.9)	4.6 s (2.1)

*Standard deviations are included in parentheses*.

We then analyse these results in more detail by breaking them up into RT_utterance_ and RT_question_. We ran the same ANOVA analysis, first using RT_utterance_ as the dependent variable and then using RT_question_ as the dependent variable. With regard to RT_utterance_, it showed no main effect of Face Condition [*F*_(1, 288)_ = 1.09, *p* = 0.30], no main effect of Response Type [*F*_(1, 288)_ = 0.00, *p* = 0.99], and no interaction effect [*F*_(1, 288)_ = 1.12, *p* = 0.29]. On the other hand, with regard to RT_question_, the ANOVA revealed a main effect of Face Condition [*F*_(1, 288)_ = 4.21, *p* = 0.04] but no main effect for Response Type [*F*_(1, 288)_ = 0.58, *p* = 0.45]. There was a tendency toward an interaction [*F*_(1, 288)_ = 3.51, *p* = 0.06].

Finally, a logistic regression analysis was conducted to predict Response Type (“Yes”/”No”) using as predictors Face Condition, RT_utterance_, RT_question_, and the interaction between Face Condition and both RT measures. A test of the full model against a constant only model failed to reach statistical significance, indicating that the predictors as a set did not distinguish between “Yes” and “No” answers (chi square = 9.16, *p* = 0.10 with *df* = 5).

#### Discussion

In line with Bonnefon and colleagues, the results suggest that in face-threatening contexts people are more likely to answer positively to the semantic compatibility question, i.e., indicating that some participants ultimately believed that *Everybody hated your speech* when told that *Some people hated your speech*. This replicates the results of a series of studies by Bonnefon and colleagues (Bonnefon et al., 2009, 2011; Feeney and Bonnefon, 2012) and confirms the robustness of this effect.

In contrast, Study 1 did not provide robust support to Bonnefon et al.'s (2011) original reaction time claim. Study 1 failed to replicate their significant interaction when considering the utterance and question together, though it did reveal a tendency in the expected direction (*p* = 0.10). That is, participants who answered “Yes” tended to be slower overall, and only in the face-threat condition.

However, thanks to our design, we could further investigate potential reaction time differences at the presentation of the scalar utterance (RT_utterance_) and of the semantic compatibility question (RT_question_). Contrary to predictions based on Bonnefon et al.'s (2011), we did not find any significant reaction time difference for RT_utterance_. We did find a significant effect of condition for RT_question_, with slower responses in the face-threat condition than in the face-boost condition. Furthermore, in line with predictions based on Mazzarella (2015), the interaction effects suggest that “Yes” answers (5.2 s) tend to take longer than “No” answers (4.6 s) in the face-threat condition (the latter being comparable with “Yes” and “No” answers in the face-boost condition−4.4 and 4.6 s respectively).

In sum, Study 1 provides no evidence that the scalar utterance is interpreted at different speeds across the two conditions. On the other hand, the data do suggest that the process of epistemic evaluation, which operates when answering the semantic compatibility question, is the source of reaction time differences. These results confirm our hypothesis that the process of epistemically evaluating the piece of incoming information plays a crucial role in Bonnefon and colleagues' task, and that this needs to be distinguished from the process of interpreting the scalar utterance. Epistemic assessment may lead to the rejection of the scalar inference, particularly in these face-threatening contexts (because of politeness considerations). Given that the rejection of the executed scalar inference would ultimately provide a “Yes' answer, this would explain why the percentage of “Yes” answers to the semantic compatibility question increases in the face-threat condition, and why they tend to be longer than “No” answers.

### Study 2

Study 2 aimed at increasing the likelihood that participants would derive the scalar inference. To achieve this, we manipulated the background scenario in the following two ways: (i) we increased the relevance of the scalar inference by introducing a slightly different implicit question that puts the focus on the delivery of the speech (*You tell Denise that you would like to know the audience's reaction* instead of *You tell Denise that you are thinking about giving the same speech to another group*) and; (ii) we explicitly characterize the speaker (Denise) as knowledgeable with regard to the question at issue (i.e., the so-called “competence assumption” is made clear, see, e.g., Sauerland, 2004; Breheny et al., 2013). See Appendix A for a thorough comparison with Bonnefon et al.'s (2011) material.

As discussed in the literature, the scalar inference is facilitated when the speaker is assumed to be in a position to know the entire situation, i.e., that she is in a position to know that the stronger alternative is false. That is, an utterance of *Some people loved your speech* would be taken to license the scalar inference *Not everybody loved your speech* if one could assume that the speaker knows everybody's opinion about the speech and is in a position to rule out the possibility that everybody loved it. Bonnefon et al.'s scenarios do not clearly attribute such knowledge to the speaker. In fact, it is not clear whether in the original paradigm Denise is at all aware of the opinion of all the other members of the audience. This leaves open the possibility that some participants might have assumed that Denise did not know everyone's opinion, and so the listener is arguably not in a position to know whether a scalar inference is called for. Our manipulation (ii) should overcome this limitation. It follows that if the manipulations in (i) and (ii) are effective, the percentage of “Yes” answers to the semantic-compatibility question in the face-boost condition should drop from Study 1 to Study 2 because the scalar inference is called for with greater confidence. Assuming that our manipulations do facilitate the derivation of the scalar inference, we will be in a better position to analyze the role played by the scalar utterance and the semantic compatibility question with respect to the reaction time effects.

#### Material and methods

We recruited 398 participants through Amazon Mechanical Turk (230 men, 168 women, mean age 32.6, *SD* = 10.5). Each participant read a version of the Speech vignette either in the face-boost version or in the face-threat version.

(6a) Imagine you gave a speech at a small political meeting. You are discussing your speech with Denise, who was also there. There were 6 other people in the audience that day and you know that Denise spoke with all of them about it later. You tell Denise that you would like to know the audience's reaction.
(6b) Hearing this, Denise tells you that “Some people hated your speech.”

As in Study 1, the texts corresponding to (6a) and (6b) were displayed separately in two steps and the face-boost version presented the scalar utterance with *Some people loved your speech*. After reading the story, participants were presented the semantic compatibility question:

(6c) Given what Denise told you, do you think that it is possible that everybody hated [loved] your speech?

There was no conversational implicature question.

This study was carried out in accordance with the Décret n° 2017-884, whose article R. 1121- 1. -IId indicates that such research does not have to receive IRB approval in France. All subjects gave written informed consent in accordance with the Declaration of Helsinki.

#### Analysis

Using the same criteria as Study 1, we excluded 87 participants because of the presence of unnecessary clicks, 2 participants because their RTs betrayed a clear lack of attention and 15 participants as outliers. Our final sample included 294 participants.

#### Results

##### Semantic compatibility question

Responses to the semantic compatibility question (see Table 6) reveal that few participants in the face-boost condition thought it was possible that everyone loved the speech, while in the face-threat condition, participants remained split between the two answers (Fisher exact test, *p* < 0.001, *OR* = 5.7). The minor modifications in Study 2—rendering the speaker omniscient and the utterance more relevant—prompted the anticipated result. The result is that the face-threat/face-boost distinction is much clearer here than in Study 1.

**Table 6:** Percentage of “Yes” responses to the semantic compatibility question in Study 2.

	**Hearing this, Denise tells you that**	
	“Some people hated your speech” **Face-threat condition**	“Some people loved your speech” **Face-boost condition**
**SEMANTIC COMPATIBILITY QUESTION**		
do you think that it is possible that everybody hated [loved] your speech?	45%	12.5%

*For each cell, the complement corresponds to “No” responses*.

##### Reaction times

We first determined whether the combined RTs prompted effects reminiscent of Bonnefon et al. (2011). Table 7 displays the RTs collected. A two-way ANOVA was conducted that examined the effect of Response Type (“Yes”/”No”) and Face Condition (Face-boost/Face-threat) on the combined response times (RT_utterance_ + RT_QUESTION_). It revealed no significant effects (main effect of Face Condition [*F*_(1, 290)_ = 2.71, *p* = 0.10], main effect of Response Type [*F*_(1, 290)_ = 1.14, *p* = 0.29], interaction effect [*F*_(1, 290)_ = 1.02, *p* = 0.32].

**Table 7:** Mean response time (in seconds) for “Yes” and “No” answers to the semantic compatibility question, in the face-boost and in the face-threat conditions (Study 2).

	**Face-threat**	**Face-boost**
**SCALAR UTTERANCE + SEMANTIC COMPATIBILITY QUESTION**		
“Yes”	8.4 s (3.2)	7.2 s (2.5)
“No”	7.7 s (3.2)	7.5 s (3.2)
**SCALAR UTTERANCE (COMPREHENSION ASSESSMENT STAGE)**		
“Yes”	2.8 s (1.2)	2.8 s (0.9)
“No”	3.1 s (1.3)	2.9 s (1.3)
**SEMANTIC COMPATIBILITY QUESTION**		
“Yes”	5.6 s (2.4)	4.4 s (1.9)
“No”	4.6 s (2.3)	4.6 s (2.5)

*Standard deviations are included in parentheses*.

We then performed the same ANOVA using RT_utterance_ as a dependent variable and found no significant effects (main effect of Face Condition [*F*_(1, 290)_ = 0.05 *p* = 0.83], main effect of Response Type [*F*_(1, 290)_ = 1.17, *p* = 0.28], interaction effect [*F*_(1, 290)_ = 0.62, *p* = 0.43]. However, when we used RT_question_ as dependent variable, the ANOVA revealed a main effect of Face Condition [*F*_(1, 290)_ = 4.91, *p* = 0.03], a main effect of Response Type [*F*_(1, 290)_ = 5.06, *p* = 0.03], and a tendency toward an interaction [*F*_(1, 290)_ = 3.18, *p* = 0.08].

Finally, a logistic regression analysis was conducted to predict Response Type (“Yes”/”No”) using as predictors Face Condition, RT_utterance_, RT_question_, and the interaction between Face Condition and both RT measures (Face Condition^*^RT_utterance_ and Face Condition^*^RT_question_). A test of the full model against a constant only model was statistically significant, indicating that the predictors as a set reliably distinguish between “Yes” and “No” answers (chi square = 53.46, *p* < 0.001 with *df* = 5). A Wald test showed that the interaction Face Condition^*^RT_question_ significantly predicts response type [Wald (1) = 4.7, *p* = 0.03]. An increase of 1 s in response time for answering the semantic compatibility question and being in the face-threat condition makes participants 1.4 times more likely to answer “Yes.” By contrast, neither the interaction Face Condition^*^RT_utterance_ nor any of the factors individually (Face Condition, RT_utterance_ and RT_question_) turned out to be significant predictors [Face Face Condition^*^RT_UTTERANCE:_ Wald (1) = 2.1, *p* = 0.15, Condition: Wald (1) = 2.3, *p* = 0.13, RT_utterance_: Wald (1) = 0.05, *p* = 0.82, RT_question_: Wald (1) = 0.04, *p* = 0.84]. It is important to note that, while the interaction Face Condition^*^RT_utterance_ is not statistically significant, the tendency goes in a direction opposite to the Face Condition^*^RT_question_ interaction effect. That is, an increase of 1 s in reading time for the scalar utterance and being in the face threat condition makes participants 1.4 times *less* likely to answer “Yes” to the semantic compatibility question [*OR* = 0.7]. In other words, those subjects who spend more time reading the scalar utterance tend to respond negatively later to the semantic compatibility question.

#### Discussion

In line with our expectations, modifying the task so that it maximizes the coherence between the background story and the utterance while also presenting Denise as omniscient about the relevant group of people affected the percentage of “Yes” and “No” answers to the semantic compatibility question. Compared to the task in Study 1, Study 2 presents higher rates of negative responses to the semantic compatibility question in the face-boosting condition. That is, by ensuring the relevance of the scalar inference, we replicated and sharpened the results of Study 1 (in line with Bonnefon and colleagues' work).

As in Study 1, we found no evidence of reaction time differences with respect to the presentation of the scalar utterance (RT_UTTERANCE_), as should be predicted by Bonnefon et al. (2011). In fact, our results suggest that, if anything, being in the face-threat condition and displaying longer reading times for the scalar utterance is less likely to produce “Yes” answers to the semantic compatibility question. This is in direct contrast with Bonnefon et al.'s claim that longer reaction times for “Yes” responses in the face-threat condition are linked to the extra costs imposed by politeness considerations on the processing of the scalar utterance. Our results show that, on the contrary, slow “Yes” responses are due to the process of epistemic assessment, which takes exceptionally longer in the face-threat condition when participants answer the semantic compatibility question. This interaction was indeed the only significant predictor of Response Type to the semantic compatibility question.

## General discussion

Bonnefon and colleagues investigate the effect of politeness in the computation of scalar inferences. Based on their findings, they suggest that politeness “appears to add a layer of complexity to the usual processes involved in the interpretation of *some*” (Bonnefon et al., 2011, p. 3393). Specifically, they claim that the effect of politeness is two-fold: on the one hand, it blocks the derivation of the scalar inference in face-threatening contexts and, on the other, it makes the semantic interpretation of “some” (*at least some and possibly all*) appear to be an effortful step. While their (2011) findings—which reveal slowdowns when giving positive responses to the semantic compatibility question—are consistent with their account, their analysis is based on a task whose dependent measure does not isolate the scalar utterance. Their task involves reading a scalar utterance that (a) serves as an indirect response to a more pressing question and that; (b) is then re-assessed meta-linguistically in the task's semantic compatibility question, which is its real dependent measure.

The main aim of our studies was to reevaluate Bonnefon et al.'s findings and to reanalyse the task through the lens of *epistemic vigilance*. While comprehension involves the pragmatic ability to infer the speaker's meaning from linguistic and contextual cues, epistemic assessment involves what Sperber et al. (2010) call a capacity for epistemic vigilance, which enables hearers to avoid being accidentally or intentionally misinformed. Sperber et al. (2010) have suggested that there are two main factors affecting the believability of a piece of communicated information: the reliability of its source and the believability of its content. Our hypothesis—based on Mazzarella (2015)—was that epistemic vigilance toward the source may affect the believability of scalar inferences in face-threatening contexts. In such contexts, participants recognize that the speaker is trying to be nice and polite (by allowing the listener to generate a reading that can be glossed as *Some but not all the people hated your speech*); however, participants also recognize that it is probable that the speaker's comment is perhaps well meaning but that it is not entirely honest and, consequently, they judge part of what she communicates as not true, so they do not accept it. While participants are likely to conclude that *everyone hated the speech*, participants answer affirmatively to the semantic compatibility question because they are rejecting the speaker's communicated information. This shows how there is a distinction to be made between what is communicated in a comprehension stage and what is believed (or not) in an epistemic assessment stage. We have argued that responses in this task are due to reactions at the epistemic assessment stage; according to Bonnefon et al., the task's question is merely a measure of scalar inference-making.

In order to adjudicate between the two competing claims, we experimentally separated the task's scalar utterance from the dependent measure—responses to the semantic compatibility question. We thus (1) separated the reading of the scalar utterance from the reading/responding to the test question and we (2) made the utterance more relevant to the participant's task wherever possible. With respect to (1) we took separate reading time measures of the scalar utterance and the response to the semantic compatibility question (while also adding the two together) in both of our studies. Overall, we find no evidence that the scalar utterance is interpreted at different speeds across the two (face-threat vs. face-boost) conditions. Our data suggest, instead, that the process of epistemic evaluation, which operates when answering the semantic compatibility question, is the source of the reaction time differences. This undermines any claim that suggests that participants slow down while *drawing* a semantic reading of the utterance on line.

Overall, our results show that there is a range of responses to scalar utterances. As prior studies have shown, they are often not drawn at all. We see evidence of that here, through the large minority of participants in the face-boost condition of Study 1 who give Yes-Yes responses (in line with findings from Degen and Tanenhaus, 2015). As prior studies have also shown, including those generated by the current paradigm, participants can be encouraged to generate scalar enrichments (see Study 2) once the competence assumption can be more confidently endorsed. This can be seen through the high percentage of participants who respond negatively to the semantic compatibility question. The added value of the current work is that it shows that one can experimentally capture a third process. That is, work with the current paradigm shows that people often make the scalar enrichment because it is part of a speaker's communicated meaning but that eventually a listener can reassess that communicated information and reject it. In the current case, this is due to effects of politeness. We suggest that politeness affects the process of epistemically evaluating a piece of incoming information as presented by the speaker. When the speaker is perceived as motivated by politeness concerns, her reliability as a trustworthy informant becomes questionable. As a consequence, addressees are more likely to reject what the speaker is communicating to them (e.g., a pragmatically enriched scalar utterance). It is sensible to assume that taking these concerns into consideration requires additional processing effort (reflected in longer reaction times at the epistemic assessment stage).

These data, all inspired by Bonnefon et al.'s paradigm, open up an interesting direction of research within the field of experimental pragmatics. Crucially, they highlight the importance of taking into consideration the cognitive distinction between comprehension and acceptance. This distinction, which has long been acknowledged in the philosophical literature thanks to the seminal work of Austin and Grice, have been neglected in the experimental pragmatics literature so far. The challenge for the future is to devise new paradigms to study comprehension and epistemic assessment as two distinct components in the process of forming beliefs via testimony.

## Author contributions

DM devised the project and the main conceptual ideas. DM and IN designed the studies. DM and ET implemented the studies and ET carried out the analysis of the results. DM wrote the manuscript with input from IN and HM. All authors helped shape the research and discussed the results.

### Conflict of interest statement

The authors declare that the research was conducted in the absence of any commercial or financial relationships that could be construed as a potential conflict of interest.

## Footnotes

^1^Mazzarella (2015) suggests that the speaker would go as far in her face-saving work as “plausible deniability” allows (Lee and Pinker, 2010). That is, the speaker might not want to commit to a blatantly false statement, but rather communicate a falsity only implicitly (as in the case of the scalar inference *Not all people hated your speech* in a situation in which the speaker knows that everybody hated the speech).

^2^On the general reliability of MTurk data see, e.g., Capaldi (2017) and Zwaan et al. (2017). For evidence concerning the reliability of on-line reaction time data, see Crump et al. (2013).

^3^This “clicking criterion” was introduced in order to make sure that reaction times would reflect *spontaneous* interpretation or epistemic assessment of the scalar utterance. By eliminating errant or superfluous clicks we could be assured that slowdowns (or lack of thereof) would reflect an immediate response and not, for example, an inability to find the relevant answer box, a temporary interruption of the task, or a second thought (with no way to distinguish among these possibilities).

## Appendix A

The table below displays the Speech story in the original version from Bonnefon et al. (2011) (translated from Dutch), as well as in the modified versions of Study 1 and Study 2. Relevant changes to the original story are in bold. Horizontal lines indicate where participants were asked to advance the text.

**Table:**

**Bonnefon et al., 2011**	**Study 1**	**Study 2**	
Imagine you gave a speech at a small political rally. You are discussing your speech with Denise, who was in the audience. There were 6 other people in the audience. You are considering whether to give this same speech to another audience.	Imagine you gave a speech at a small political meeting. You are discussing your speech with Denise, who was also there. There were 6 other people in the audience that day. You tell Denise that you are thinking about giving the same speech to another group.	Imagine you gave a speech at a small political meeting. You are discussing your speech with Denise, who was also there. There were 6 other people in the audience that day and **you know that Denise spoke with all of them about it later. You tell Denise that you would like to know the audience's reaction**.	
Hearing this, Denise tells you that “Some people loved/hated your speech.” Given what Denise told you, do you think that it is possible that everybody loved/hated your speech?	Hearing this, Denise tells you that “Some people loved/hated your speech.”	Hearing this, Denise tells you that “Some people loved/hated your speech.”	RT_utterance_
	Given what Denise told you, do you think that it is possible that everybody loved/hated your speech?	Given what Denise told you, do you think that it is possible that everybody loved/hated your speech?	RT_question_
	**Given what Denise tells you, do you think that she means that you should give the speech again to another group?**		
